# Supplementary material for: Single Radiotherapy Fraction with Local Anti-CD40 Therapy Generates Effective Abscopal Responses in Mouse Models of Cervical Cancer
Source: Cancers (Basel). 2020 Apr 22;12(4):1026. doi: 10.3390/cancers12041026 (PMC7226489; doi:10.3390/cancers12041026)
Supplement: Supplementary file 1 [file cancers-12-01026-s001.pdf]

Supplementary Material: Single Radiotherapy Fraction with Local Anti-CD40 Therapy Generates Effective Abscopal Responses in Mouse Models of Cervical Cancer

Jana Wood, Sayeda Yasmin-Karim, Romy Mueller, Akila N. Viswanathan and Wilfred Ngwa

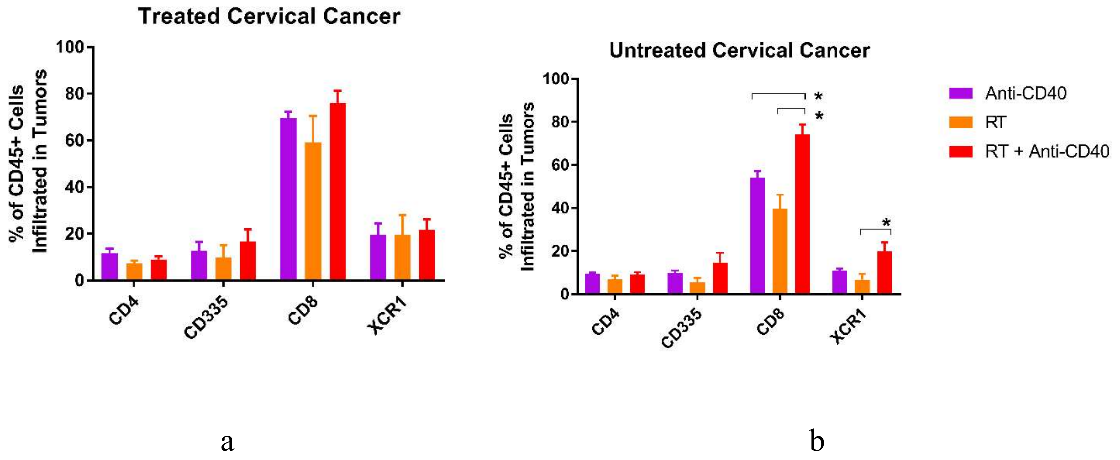

Figure S1. Flow cytometry analysis of treated and untreated mouse cervical tumors. C57BL/6 mice bearing TC-1 tumors on both flanks were treated when tumors reached 4 mm in size. Only the right tumors were treated with a single dose of 6 Gy and/or intratumoral injection of anti-CD40 (20 μg/tumor/mouse). The left tumors remained untreated. All tumors were harvested 14 days after the treatment for immune profiling focusing on helper T-cells (CD4), cytotoxic T-cells (CD8), NK-cells (CD335) and cDC1 dendritic cells (XCR1). (**a**) Immune populations infiltrating treated tumors and (**b**) untreated tumors. Significant increase of CD8+ T cells and dendritic cells populations in untreated tumors support the observation of the abscopal effect in combination treatment groups (RT + anti-CD40) compared to groups only treated with radiotherapy (RT) or anti-CD40. Error bars are SEM. n = 3 mice/group, * $p < 0.05$.

© 2020 by the authors. Licensee MDPI, Basel, Switzerland. This article is an open access article distributed under the terms and conditions of the Creative Commons Attribution (CC BY) license (http://creativecommons.org/licenses/by/4.0/).
